# Imaging the PM/AICD patient; Is it beneficial to the final diagnosis?

**DOI:** 10.1186/1532-429X-17-S1-T9

**Published:** 2015-02-03

**Authors:** June A Yamrozik, Mark Doyle, Ronald B Williams, Geetha Rayarao, Diane V Thompson, Moneal Shah, Huma Samar, Robert W Biederman

**Affiliations:** 1 Cardiac MRI, Allegheny General Hospital, Pittsburgh, PA, USA; 2 Cardiology, VA Medical Center, Los Angeles, CA, USA

## Background

Pacemaker/ AICD imaging is currently being done on patients in the MRI environment. A vigilant team consisting of the (Cardiologist, EP staff, and technologists) with close patient monitoring and supervision is making such an unmentionable procedure a success. However, is the diagnosis from imaging these patients adding valuable irrefutable information to merit such a risk?

### Hypothesis

We propose that MRI imaging patients with a pacemaker is crucial to the existing diagnosis and in some instances modifies diagnosis and patient management.

## Methods

A total of 100 patients were imaged on a GE CV/i Excite Version 12,1.5 T system (GE, Milwaukee, WI). 17 patients had an AICD, 10 patients had an AICD/Pacemaker, 5 patients had a single pacemaker lead, 8 REVO pacemakers and 60 patients had a complete pacemaker implantation. A specific criteria was followed for all the patients undergoing this procedure to scrutinize if the final diagnosis provided additional information in patient care. A checklist of three questions were gathered and answered after the final interpretation of the MRI.

1) Does the diagnosis change?

2) Does the MRI provide additional information to the existing diagnosis?

3) Does patient management change?

If yes was answered any of the above questions it was considered that the MRI scan was of value to patient diagnosis.

## Results

All patients completed the procedure with no adverse events and the pacemaker was interrogated after the procedure by EP Lab and the Cardiologist. Regarding the population, of the 100 patients imaged, 71 (71%) were neurology cases, 24 (24%) were cardiac/vascular and 5 musculoskeletal(5%).

After reviewing the results from the 71 neurology cases 17 (24%) out of the 71 showed that the MRI provided additional information and changed the original diagnosis and in turn their course of medical treatment. 37 patients (52%) provided additional information and therefore a total of 54 patients (76%) showed that the MRI scan was of value to the final diagnosis. 17 (24%) out of the 71 patients confirmed original diagnosis. The 24 cardiac cases showed in 5 patients (21%) the MRI provided additional information to change the original diagnosis and also patient management. 18 (75%) showed that extra information was gathered and 1 (4%) confirmed the original diagnosis. In essence, 96% of the Cardiac population benefited by having this study done. The 5 musculoskeletal demonstrated that 4 (80%) provided additional information and 1 (20%) changed patient management

## Conclusions

The use of PM/AICD imaging in MRI remains controversial but with a cautious cardiac team increased confidence in its use is found. Herein, we show that MRI procedures on carefully selected patients with pacemakers/AICD's are beneficial and substantially add valuable irrefutable information to patient diagnosis and management. We propose that not only are Pacemakers/AICD's no longer *forbidden* in the MRI environment but they can be markedly efficient with life-altering and life-saving consequences.

## Funding

Internal.

